# Economic burden of breast cancer: a case of Southern Iran

**DOI:** 10.1186/s12962-023-00470-8

**Published:** 2023-08-29

**Authors:** Faride Sadat Jalali, Khosro Keshavarz, Mozhgan Seif, Majid Akrami, Abdosaleh Jafari, Ramin Ravangard

**Affiliations:** 1 Student Research Committee, School of Health Management and Information Sciences, Shiraz University of Medical Sciences, Shiraz, Iran; 2 Health Human Resources Research Centre, School of Health Management and Information Sciences, Shiraz University of Medical Sciences, Shiraz, Iran; 3 Non-communicable Disease Research Center, Department of Epidemiology, School of Health, Shiraz University of Medical Sciences, Shiraz, Iran; 4 Breast Diseases Research Center, Shiraz University of Medical Sciences, Shiraz, Iran

**Keywords:** Cost of illness, Breast neoplasms, Direct service costs, Indirect expenditures

## Abstract

**Background:**

Breast cancer is one of the main causes of death from cancer around the world, imposing a significant economic burden on the families and healthcare system. The present study aimed at determining the economic burden of breast cancer in the patients referred to the medical centers in Fars province in southern Iran in 2021.

**Methods:**

This cross-sectional study is a partial economic evaluation and a cost-of-illness study with a bottom-up and prevalence-based approach, conducted in Fars province in southern Iran in 2021 from the societal perspective. A total of 230 patients were randomly included in the study, and a researcher-made data collection form was used to collect the required data. The data on direct medical costs were collected using the information on patients’ medical and financial records. On the other hand, the data on direct non-medical and indirect costs were obtained using self-reports by the patients or their companions. The Excel 2016 software was used to analyze the collected data.

**Results:**

The results showed that the annual cost of each breast cancer patient in the studied sample was 11,979.09 USD in 2021. Direct medical costs accounted for the largest share of costs (70.69%, among which the cost of radiotherapy was the highest one. The economic burden of the disease in the country was estimated at 193,090,952 USD.

**Conclusions:**

In general, due to the high prevalence of breast cancer and the chronicity of this disease, its medical costs can impose a heavy economic burden on society, the health system, the insurance system, and patients. Thus, in order to reduce the costs, the following suggestions can be offered: the use of advanced radiotherapy techniques, increasing the insurance coverage of required services, establishing low-cost accommodation centers near medical centers for the patients and their companions, providing specialized medical services for the patients in towns, using the Internet and virtual space to follow up the treatment of the patients, and carrying out free screening programs and tests for faster diagnosis of the infected patients and susceptible or exposed people.

## Background

Breast cancer is a type of cancer that occurs when breast tissue cells grow and divide in an uncontrolled way, forming a mass of tissue called a tumor [1, 2]. Breast cancer is the first cause of death from cancer in women worldwide [3]. According to the latest report of the World Health Organization (WHO), about 2.3 million new cases of breast cancer were registered in 2020, and 685,000 deaths from this disease were reported in the same year [4]. As stated by the International Agency for Research on Cancer in 2022, the number of patients is expected to reach 3.19 million by the end of 2040 [5]. The number of new cases registered in 2020 was as follows: 491,691 in the Americas, 576,337 in Europe, 119,452 in Eastern Mediterranean, 298,445 in Southeast Asia, 635,439 in Western Pacific, and 139,477 in African countries [6]. With 16,967 cases of breast cancer in 2020, Iran had the highest rate of the disease among all types of cancer, and 4,810 patients died in that year [6].

Breast cancer can markedly affect the health of patients and impose a great economic burden on their families and society. Moreover, due to the poor awareness of screening services, most women with breast cancer are diagnosed at a late stage, after the optimal treatment time has ended. Thus, when cancer progresses to its last stage, higher costs and poor response to treatment along with an increase in economic burden are imposed on the whole family [7].

On the other hand, a cost of illness (COI) study is defined as the determination of the value of the resources consumed or lost as a result of a health problem, which includes the costs to the health sector (direct costs), the value of reduced or lost productivity by the patient (indirect costs), and the cost of pain and suffering (intangible costs) [8]. However, since intangible costs are rarely measured in COI studies due to measurement problems [9], the present study, too, mainly focused on the first two cost categories.

Health policymakers and planners are interested in understanding the economic burden of breast cancer for optimal allocation of health resources and cost estimation [10]. Researchers are also expected to focus on key diseases, that impose a high economic burden [11], one of which is breast cancer that is the second most expensive after colorectal cancer, costing New Zealand $126.7 million annually [12]. In the United States, the costs of breast cancer health care were estimated at approximately $20 billion in 2020 [13], and in South Korea and Spain, the estimated costs of breast cancer diagnosis and treatment were $940.75 million and €518 million in 2013, respectively [14, 15]. Although the regional estimation of the economic cost of breast cancer is necessary to prevent and control the disease in developing countries, there are few studies on the estimation of the economic burden of breast cancer in these countries [10]. For example, a study in Saudi Arabia reported that the average cost of breast cancer in 2018 was 14,249 USD [16]. Like in other developing countries, the incidence of breast cancer in Iran has increased and imposed a significant financial burden on the families and healthcare system [10, 17]. In this regard, Askarzadeh et al. (2019) in eastern Iran found that the per capita cost of hospitalization for a breast cancer patient was 243.39 USD in 2018 [18], and Afkar et al. (2021) estimated that the average hospital costs in private and public centers were 10,050 and 3,960 USD, respectively, and the average total indirect cost was 22,350 USD in 2017 [17]. In their study in 2010, Dorudi et al. (2015) estimated the economic burden of breast cancer at 947,375,468 USD [10]. On the other hand, Davari et al. (2013) estimated that the average monthly direct costs per patient in stages I to IV were 222.17, 224.61, 316.51, and 828.52 USD, respectively, from 2005 to 2010 [19].

To the best knowledge of the researchers, none of the studies conducted in Iran had comprehensively investigated all components of breast cancer costs. Therefore, the current study aimed at determining the economic burden of breast cancer in the patients referred to medical centers in Fars province in southern Iran in 2021.

## Methods

### Design and population

This partial economic evaluation and COI study was conducted as a cross-sectional study over a one-year period from March 2021 to March 2022. In the present study, the COI study refers to the value of resources expended or lost due to a health problem, which includes direct costs (consisting of healthcare costs and non-healthcare costs) incurred by the health system, society, family and individual patient, and indirect costs resulting from productivity losses due to morbidity and mortality, borne by the individual, family, society, or the employer [20, 21].

The research population included all patients with breast cancer in Fars province, which is the fourth most populous province of Iran. Based on the findings of Davari et al. (2013) [19] and using the following formula, assuming S = 0.63, d = 0.06, and α = 0.05, the sample size was determined as 230 breast cancer patients:

$$n=\frac{{z}_{1-\frac{\alpha }{2}}^{2}\times {s}^{2}}{{d}^{2}}$$

In order to select the samples through the simple random sampling method, breast cancer patients from all medical centers providing diagnostic and treatment services were selected using the list of the patients provided by the Cancer Registry Research Center, and their cost data was examined. The criteria for the inclusion of patients in the study were living in Fars province until the end of the study period, consent to participate in the study, and receiving continuous treatment as an outpatient or inpatient.

The bottom-up approach was used to calculate the costs from the societal perspective [22]. In addition, the prevalence-based study is used when the costs of a disease over a period of usually one year are available [23].

### Data collection

To collect the required data, a data collection form was prepared using the opinions of the experts in Oncology, Health Services Management, and Health Economics. The data collection form included four sections as follows: demographic characteristics, direct medical costs, direct non-medical costs, and indirect costs. It is worth noting that the costs were based on the US dollar (USD), which was equal to 42,000 Rials in Iran in the study year, obtained from the website of the Central Bank of Iran (CBI) [24].

A) **Demographic characteristics**: Demographic information of the patients such as age, sex, marital status, education level, insurance coverage, and the stage of the disease were collected by reviewing the patients’ medical records and asking the patients or their companions on the telephone.
B) **Direct Medical Costs (DMC)**: The direct medical costs of each patient were collected using a researcher-made checklist and referring to the medical centers under study. The costs included physician and oncologist visits, medications and drugs, laboratory tests, radiography, radiotherapy, chemotherapy, hospitalization, etc. which were collected by reviewing the patients’ medical and financial records and asking them or their companions on the telephone. In order to find the exact price of the medicines and drugs, the researchers referred to the Deputy of Food and Drug of Shiraz University of Medical Sciences. In addition, in order to calculate the direct medical costs, the average total annual direct medical cost for each patient was calculated as follows:
  The total annual direct medical cost of each patient = (average number of visits per year × visit tariff) + (average number of diagnostic-therapeutic services per year × tariff for each service) + (average number of hospitalizations per year × tariff for each day of hospitalization) + (the number of medications and drugs prescribed in a treatment period × the cost of each medication and drug unit).
C) **Direct Non-medical Costs (DNMC)**: To estimate the direct non-medical costs during the study period, in addition to the self-reports obtained from patients or their companions, the approved government tariffs for the costs of accommodation, food, and transportation were used. Given that most of the patients referred to the medical centers were living far away from the service providers, items such as travel costs of the patients and their companions to the medical centers to receive services and the accommodation and food costs were considered as the components of direct non-medical costs. The average total annual direct non-medical cost per breast cancer patient was obtained as follows:
  Average cost per patient = number of visits to receive medical services per year × cost of each visit.
D) **Indirect Costs (IC)**: The data on the indirect costs were collected through telephone interviews with the patients who received inpatient and outpatient services from the studied medical centers during the study period or their companions. The indirect costs included the costs of productivity loss due to the disease (morbidity costs) and due to premature death (mortality costs). To calculate the indirect costs, the human capital approach was used [21, 25]. The individuals’ wages were used to calculate lost income [26]. The minimum salary in the study year was considered as the individuals’ salary level, which was equivalent to 21.07 USD per day, and according to the approval of the Ministry of Labor, Welfare, and Social Security, every 8 h was determined to be one working day [27]. The potential productivity loss due to outpatient visits and hospitalization was calculated for each patient. To calculate the cost of premature death, the age of any deceased patient was deducted from the standardized life expectancy for the country (74.6 years for men and 76.9 years for women) [28] to obtain the time lost. For deceased patients, the potential productivity loss was calculated based on their occupation and salary, and a 15% rate was added to their salary every year. A discount rate of 5.8% was applied to calculate the time value of money because future values are usually less valuable than present values [29]. Finally, the lost time was multiplied by the calculated amount of wages to obtain the cost of premature death due to the disease [30, 31]. It is important to note that in the present study, there was no premature death due to the disease, and therefore, there was no calculation of mortality costs.

### Calculation of economic burden of breast cancer

The economic burden of all breast cancer patients in Iran was calculated using the following formula after estimating the average direct and indirect costs for each patient in the present study and the prevalence rate of breast cancer patients in the country:

Economic burden = Total cost (Direct Medical Cost + Direct Non − medical Cost + Indirect Cost) ∗ the estimated number of breast cancer patients in Iran [32].

### Statistical analysis

The Excel 2016 software was used to analyze the collected data.

### Ethical considerations

This study was approved by the Ethics Committee of Shiraz University of Medical Sciences (Code: IR.SUMS.REC.1400.052). The patients were free to choose to participate in the study, and once the objectives of the research were explained to them, their written informed consent was obtained. The questionnaires and checklists were completed anonymously and the patients were assured of the confidentiality of their answers to the questions. It should be noted that to comply with ethical considerations and the confidentiality of patient information, the patients were distinguished by the codes at the top of the data collection form.

## Results

### Demographic characteristics

As shown in Table 1, all the 230 breast cancer patients studied were female, and most of them were married (77%), in the age range of 42–64 years (63%), with an under-Diploma degree (60%), with a body mass index (BMI) of 25-29.9 (52.2%), with no history of smoking (89.6%), with no supplementary health insurance coverage (53.1%), with a monthly income of 1429–1905 USD (55.6%), non-native (65.65%), and in the third stage of the disease (44.8%). Besides, 78.3% of the patients had simultaneously used public and private centers to receive diagnostic and treatment services.

**Table 1:** Demographic Characteristics of the studied breast cancer patients (n = 230)

Characteristics		Number of patients (%)
Sex	Male	0 (0)
	Female	230 (100)
Marital status	Married	177 (77)
	Single	53 (23)
Age groups (years)	28–42	58 (25.2)
	42–64	145 (63)
	64≤	27 (11.8)
Education level	Illiterate	6 (2.6)
	Lower than diploma	138 (60)
	Academic degrees	86 (37.4)
BMI	< 18.5	2 (0.9)
	18.5–24.9	58 (25.2)
	25-29.9	120 (52.2)
	30<	50 (21.7)
Smoking	Yes	24 (10.4)
	No	206 (89.6)
Type of medical center	Public	50 (21.7)
	Public- Private	180 (78.3)
Having supplementary health insurance coverage	Yes	108 (46.9)
	No	122 (53.1)
Monthly Income (USD)	< 952	11 (4.8)
	952–1429	49 (21.3)
	1429–1905	128 (55.6)
	> 1905	42 (18.3)
Habitation status	Non-native	151 (65.65)
	Native	79 (34.35)
Stages	I	9 (4)
	II	58 (25.2)
	III	103 (44.8)
	IV	60 (26)

### Direct and indirect costs

The results of calculating direct medical, direct non-medical, and indirect costs for each breast cancer patient are shown in Table 2. As presented, the results showed that the highest average cost per patient was related to direct medical costs (70.69%). In addition, the highest average direct medical, direct non-medical, and indirect costs for each patient were related to the costs of radiotherapy services (39.67%), transportation of the patients and their companions (39.09%), and absenteeism of the patients (52.07%), respectively.

**Table 2:** Average annual direct medical, direct non-medical, and indirect costs per studied breast cancer patient (USD)

	Type of costs	Mean Costs by Stage				Total Mean ± SD	Median	%	% of total costs
		I	II	III	IV				
**Direct medical costs**	Physicians and oncologist visits	164.68	213.01	258.47	270.42	226.65 ± 103	202.38	2.68	**70.69**
	Radiotherapy	674.60	2091.87	3912.28	6758.48	3359.31 ± 5133.81	2199.05	39.67	
	Chemotherapy	215.61	764.57	1050.11	1198.81	802.78 ± 857.05	857.14	9.48	
	Radiography	386.51	536.45	546.23	537.22	501.60 ± 289.44	476.19	5.92	
	Physiotherapy	0	8.21	27.62	70.24	26.52 ± 135.76	0	0.31	
	hormone therapy	0	351.39	575.82	1000	481.80 ± 1093.05	0	5.69	
	Laboratory tests	224.87	530.79	697.87	784.03	559.39 ± 298.92	633.93	6.61	
	Lymphedema	0	16.01	89.11	67.26	43.09 ± 140.91	0	0.51	
	Hospitalization	973.65	2066.05	2448.34	2977.80	2116.46 ± 1007.85	2619.05	25.00	
	Medications and drugs	230.16	360.22	367.91	441.47	349.94 ± 312.51	321.43	4.13	
	**Total**	**2870.08**	**6938.57**	**9973.76**	**14105.73**	**8467.54 ± 6318.41**	**8644.41**	**100**	
**Direct non-medical costs**	Accommodation	26.45	345.65	697.18	769.44	459.68 **±** 1069.26	0	22.03	**17.42**
	Transportation of patients and their companions	280.42	810.86	1123.81	1048.04	815.78 ± 648.62	821.43	39.09	
	Patients and their companions’ food	207.67	612.89	982.20	796.82	649.90 ± 1041.63	476.19	31.14	
	Phone and internet calls with family	12.17	20.89	18.42	25.36	19.21 ± 27.96	11.90	0.92	
	Purchasing assistive devices	0	10.06	18.72	69.05	24.46 ± 172.65	0	1.17	
	Babysitter and housemaid	0	146.96	121.94	202.38	117.82 ± 431.05	0	5.65	
	**Total**	**526.71**	**1947.31**	**2962.27**	**2911.09**	**2086.85 ± 1969.98**	**2422.62**	**100**	
**Indirect costs**	Patient companions’ absenteeism due to patient care	502.64	1151.48	736.24	341.27	682.91 ± 1049.13	0	47.93	**11.89**
	Patients’ absenteeism due to the disease	1216.93	524.22	554.90	671.13	741.79 ± 2777.70	0	52.07	
	**Total**	**1719.57**	**1675.70**	**1291.14**	**1012.40**	**1424.70 ± 2945.82**	**4017.86**	**100**	
**Total Cost**		**5116.36**	**10561.58**	**14227.17**	**18029.22**	**11979.09 ± 7777.68**	**11893.45**		**100**

### Economic burden of breast cancer in Iran

Considering the incidence rate of breast cancer in Iran (estimated at 20.2 new cases per 100,000 persons in 2021) [8] and based on the average one-year survival rate of this disease in the country (95%) [33], and the following formula, the prevalence rate of the disease was estimated at 16,119.

Prevalence rate = Incidence rate of breast cancer * Average duration of breast cancer (estimation of 1 year) [34].

**Fig. 1:**
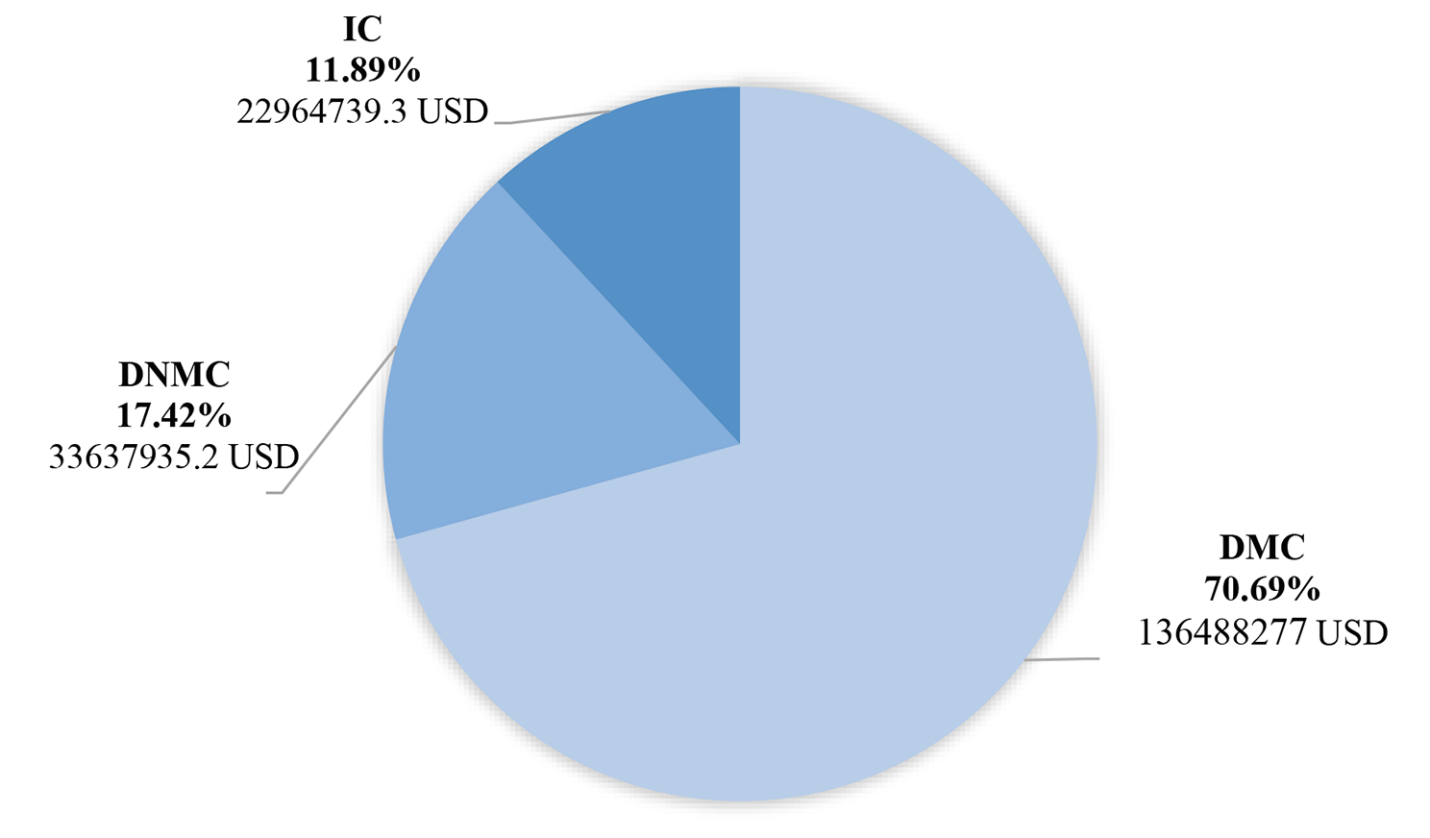
Estimation of the annual economic burden of breast cancer in Iran in 2021 from the social perspective (USD)

Then, using the average costs obtained from the results of the present study, the researchers estimated the economic burden of breast cancer in the country, the results of which are presented in Table 3. Thus, the average annual cost of each breast cancer patient in Iran was equal to 11,979.09 USD in 2021. Therefore, the economic burden of breast cancer in Iran was estimated at 193,090,952 USD. Figure 1 shows the total average of DMC, DNMC, and IC of breast cancer in Iran in 2021.

**Table 3:** Estimation of total annual costs of patients with breast cancer in Iran in 2021 (USD)

Number of patients	Direct medical costs	Direct non-medical costs	Indirect costs	Economic burden
16,119	136,488,277	33637935.2	22964739.3	193,090,952

## Discussion

Despite significant advances in breast cancer management in recent years, the incidence and mortality rates of this disease are still increasing in developed and developing countries; this has led to a significant increase in global patient-related healthcare costs [16] and has imposed a great economic burden on health care systems and communities [35]. The aim of the present study was to determine the economic burden of breast cancer in the patients referred to medical centers in Fars province in southern Iran in 2021.

The results of this study showed that most of the patients were married, in the age range of 42–64 years, with an under-Diploma degree, with a BMI of 25-29.9, with no history of smoking, with no supplementary health insurance coverage, with a monthly income of 1429–1905 USD, non-native, in the third stage of the disease, and had simultaneously referred to public and private centers to receive diagnostic and treatment services. The results of the study by Adanu et al. (2022) in Ghana on breast cancer patients showed that most of the patients were between 40 and 69 years of age, had an under-diploma education level, and more than half of them were single with an average monthly income of 370 USD [3]. In their study on breast cancer patients in Iran, Afkar et al. (2021) concluded that most of the patients had a diploma and no supplementary health insurance coverage, and their mean age at the time of diagnosis was 45.41 years [17]. In the study by Lio et al. (2018) in China, most of the breast cancer patients were 45–54 years of age, had an under-diploma education level, were in the second stage of the disease, and had referred to specialized centers for diagnostic and treatment services [7]. Likewise, most of the patients were in the second stage of the disease in the study by Blumen et al. (2015) [36].

The results of the present study showed that breast cancer had a significant economic burden on the health system and society, and direct medical costs accounted for the greatest costs. The direct medical costs of breast cancer patients alone were approximately equivalent to 1.7% of the total health expenditure (the total health budget in Iran was estimated at 39.5 billion USD according to the latest report published on the World Bank website (2019), which was equivalent to 6.71% of the gross domestic product (GDP)). The reasons for the high direct medical costs could be the great need of breast cancer patients to receive various services from numerous medical centers, the high tariffs and high prices of these services, and the long duration of treatment for this disease.

It was found out in the present study that the highest average direct medical costs per patient were related to radiotherapy services, the reason for which could be that radiotherapy was one of the expensive standard treatment interventions for these patients [37]. Given that many of the patients in this study needed to have multiple radiotherapy sessions, the cost of this part of direct medical costs was higher than others. Glynn and colleagues in their study conducted in the UK (2023) stated that although the cost of radiotherapy is very high, radiotherapy after primary surgery for breast cancer patients reduced the risk of recurrence in half for the next 10 years [38]. The results of the present study are in line with the findings of Lao et al. (2022) in New Zealand [39], Ferrier et al. (2020) in France [40], Hu et al. (2020) [41], and Sagar et al. (2017) [42] in the US, Capri et al. (2017) in Italy [43], Giordano et al. (2016) in the US [44], Ivanauskien et al. (2015) in Lithuania [45], and Bahmei et al. (2015) [46] and Yavari (2013) in Iran [47].

According to the results of the current study, the transportation of the patients and their companions accounted for the highest direct non-medical costs per patient, one of the main reasons for which was that the centers that provided diagnostic and treatment services to cancer patients were located in the capital of the province. Thus, the patients had to go to these centers from other cities to receive the services they needed. Another reason for the increased travel costs of the patients and their companions could be the need of the patients to have frequent radiotherapy, chemotherapy, and hormone therapy sessions. Furthermore, due to the side effects of chemotherapy such as nausea, anemia, and lethargy, cancer patients needed to see and consult with different specialists, causing their travel costs to increase significantly. The results of this research were in line with the studies by Adanu et al. (2022) in Ghana [3]. In the studies by Slavova et al. (2020) in Australia [48], JavanNoughabi, et al. (2018) [49] in Iran, Cheng et al. in the US (2017) [50], and Hatam et al. (2013) [51] travel costs of the patients and their companions were also considered as one of the main factors in increasing the costs of the patients, which is consistent with the results of the present study.

The highest indirect cost per patient was related to patient absenteeism, the main reason for which, as stated by some of the patients participating in this research, was that the long-term treatment of breast cancer and its complications and the need to refer to specialists for the treatment of these complications caused them to take sick leave or even lose their jobs. This had imposed huge costs on them. In their research, Mamo et al. (2017) stated that absenteeism and consequent loss of wages was an important factor in increasing the economic burden of breast cancer [52], which is similar to the results of the present study. However, the studies by Adanu et al. (2022) in Ghana, Afkar et al. (2021), JavanNoughabi, et al. (2018) [49], and Hatam et al. (2013) [51] in Iran reported that the patients’ companions’ absenteeism was the biggest driver of indirect costs [3, 17]. The reasons for the difference between the results of these studies and those of the present study could be the difference in the number of patients of working age and also the difference in the number of patients’ companions in these studies.

### Study limitations

Among the limitations of the current research was the self-report of the patients or their companions about direct non-medical and indirect costs, and as a result, forgetting some costs or mentioning them approximately and with recall bias. In addition, incomplete information in some patients’ medical records and the lack of cooperation of some patients with the researchers in providing accurate cost data could be considered as other limitations of the current study. It should be noted that in this research, due to the lack of access to some required data, it was not possible for the researchers to determine some costs such as the costs of home care and informal treatments, and intangible costs such as pain and depression.

## Conclusions

According to the findings, the highest costs of breast cancer patients were the direct medical costs (with the largest share related to radiotherapy). Some strategies can be effective in reducing the cost of radiotherapy. The use of shorter courses of radiotherapy, such as hypofractionated radiotherapy, can be as effective as longer courses, and their costs can be lower. Also, the use of advanced radiotherapy techniques such as intensity-modulated radiotherapy (IMRT) and volumetric modulated arc therapy (VMAT) can provide more accurate doses of radiation to the tumor while sparing healthy tissue, thereby reducing the risk of side effects and the need for additional treatment. Among the direct non-medical and indirect costs, the highest costs were respectively related to the patients’ and their companions’ travel as well as the patients’ absence from work. Hence, in order to reduce the costs and economic burden of breast cancer, the following suggestions can be offered: the use of flexible working hours or telecommuting for patients; increasing the insurance coverage of required services; establishing low-cost accommodation centers for the patients and their companions near medical centers; providing specialized treatment services for the patients in towns to reduce their travel costs; using the internet and virtual space to follow up the treatment of the patients in cases which there is no need to visit in person; and carrying out free screening programs and tests with the help and support of the Ministry of Health and Medical Education for faster diagnosis of the infected patients and susceptible or exposed people.

## Data Availability

The data used and analyzed in the study are available from the corresponding author on reasonable request.

## Abbreviations

WHO: World Health Organization
DMC: Direct Medical Costs
DNMC: Direct Non-medical Costs
IC: Indirect Costs
