# Correction: Economic burden of breast cancer: a case of Southern Iran

**DOI:** 10.1186/s12962-023-00478-0

**Published:** 2023-09-27

**Authors:** Faride Sadat Jalali, Khosro Keshavarz, Mozhgan Seif, Majid Akrami, Abdosaleh Jafari, Ramin Ravangard

**Affiliations:** 1 Student Research Committee, School of Health Management and Information Sciences, Shiraz University of Medical Sciences, Shiraz, Iran; 2 Health Human Resources Research Centre, School of Health Management and Information Sciences, Shiraz University of Medical Sciences, Shiraz, Iran; 3 Non-communicable Disease Research Center, Department of Epidemiology, School of Health, Shiraz University of Medical Sciences, Shiraz, Iran; 4 Breast Diseases Research Center, Shiraz University of Medical Sciences, Shiraz, Iran

**Cost Effectiveness and Resource Allocation (2023) 21:58**

10.1186/s12962-023-00470-8

Following publication of the original article, it came to the authors’ attention that the name of author Dr. Khosro Keshavarz had been incorrectly spelled: “Keshavar” had been given instead of “Keshavarz”. The name has since been corrected in the published article. The authors thank you for reading this erratum and apologize for any inconvenience caused.

